# Determination of phenolic compounds and their antioxidant activity of Iranian *Allium sativum controversum* extracts and their antimicrobial properties in fresh sausages

**DOI:** 10.1002/fsn3.3059

**Published:** 2022-10-06

**Authors:** Adeleh Madani, Nasrin Choobkar, Amir Daraei Garmakhany

**Affiliations:** ^1^ Department of Food Science and Egineering, Mahalat branch Islamic Azad University Mahalat Iran; ^2^ Plant Biotechnology Research Center Kermanshah branch, Islamic Azad university Kermanshah Iran; ^3^ Department of Fisheries, Faculty of Agriculture, Kermanshah branch Islamic Azad University Kermanshah Iran; ^4^ Department of Food Science and Technology, Toyserkan Faculty of Industrial Engineering Bu‐Ali Sina University Hamadan Iran

**Keywords:** *Allium sativum controversum* extract, antioxidant, phenolic compound, sausages

## Abstract

In this study, Iranian *Allium sativum controversum* extracts, as a valuable source of bioactive compounds such as antioxidants, extracted by solvents were analyzed. Based on the analysis of total phenolic content (TPC) and total flavonoid content (TFC) and radical scavenging activity (1,1‐diphenyl‐2‐picrylhydrazyl (DPPH)) of each extract, ethanol extracts were finally added to the sausage formulation at 0.5 and 1.5%w/v. Treatments were kept at refrigerated temperature (4–5°C) for 1, 15, and 30 days, and DPPH and microbial assays were performed on the treatments and the control samples. Experimental data were performed in a completely randomized design with the factorial arrangement. Hydroalcoholic extract had the highest total phenols and the aqueous extract of *Allium sativum controversum* showed the highest radical scavenging activity (11.85 ± 0.81 mg/g). No colony counts were observed on the first day of the coliform count. On 15 to 30 days, the control sample showed the highest count and the treatment containing *Allium sativum controversum* extract (1.5%) had the lowest coliform count. During the first month, the control sample had the highest count of *Staphylococcus aureus*. Regarding mold and yeast, a treatment containing *Allium sativum controversum* extract (1.5%) and the control sample had the lowest and highest count, respectively. The results showed that using *Allium sativum controversum* extracts and increased radical scavenging activity reduced microbial growth during the storage period.

## Graphical abstract

In this study, Iranian *Allium sativum controversum* extracts, as a valuable source of antioxidants and antibacterial compounds, were added to sausage formulation at 0.5 and 1.5%w/v. The results showed that the use of *Allium sativum controversum* extracts increased radical scavenging activity reduced microbial growth during the storage period.

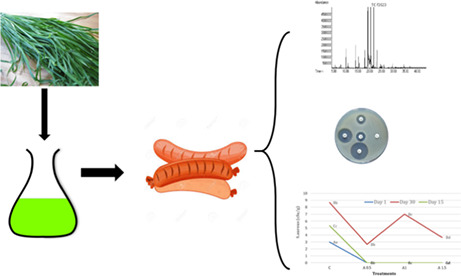

## 1 INTRODUCTION

Nowadays, chemical preservatives are being used to control the microbial population as well as to retard the oxidation reactions in food. Consumers are unsatisfied with different synthetic preservatives because of their side effects (Langroodi et al., 2018). There can be seen a global tendency toward preservation methods that are both environmental‐friendly and healthy in terms of processing, production, and preservation. Nature‐oriented methods have been prevalent in this respect, with a focus on natural additives that have long been applied in different cultures, not only promoting palatability of the treated ingredients but also lowering the perishability of foodstuff (Berger, 2009). Generally, the replacement of medicinal extracts instead with chemical preservatives is so important. It has been proved that this alternative may reduce the adverse effects of chemical preservatives (Prakash et al., 2015).

Meat and meat products are highly susceptible to both microbial growth and lipid oxidation because of their large surface to weight, leading to rapid spoilage and the development of rancid or warmed‐over flavor, respectively (Jay et al., 2008). Sausage is a product widely consumed worldwide and contains basically meat and fat (solid phase) dispersed into ice/water (liquid phase) forming a stable mass that will be submitted to a moderate heat treatment (Mercadante et al., 2010).

Microbial growth and lipid oxidation are primary factors of sausage spoilage during refrigerated storage. To extend the storage period, antimicrobial and antioxidant additives, especially of synthetic origin, are added to muscle foods (Abdel‐Salam et al., 2014). Since ancient times, herbs and spices have been added to different types of food to improve the flavor and organoleptic properties (Bagamboula et al., 2003). Also, herbal medicines have great potential in the emerging nutrition industry, because these materials are often considered foods, as well as medicines and are used in preventive and curative treatments throughout the world (Asif, 2015).

Some phenolic compounds, such as sage, rosemary, thyme, hops, coriander, tea, cloves, and basil, are known to possess antimicrobial effects against foodborne pathogens (Choobkar et al., 2022; Pateiro et al., 2021). However, their antimicrobial activity may be attributed to their ability to penetrate through bacterial membranes and inhibit the functional and lipophilic properties of the cell (Calo et al., 2015; Kalogianni et al., 2020).

Medicinal plants continue to provide valuable therapeutic agents, in both modern medicine and in traditional systems. *Alliaceae* family has been documented and valued for their spicy and medicinal qualities by many cultures around the globe for many years (Lawal et al., 2010). *Allium sativum controversum*, locally known as Varquaz, is from the Amaryllidaceae family. It is native to Iran, especially the west of Iran. *Allium sativum controversum* is a perennial herb producing a large round bulb and smells like sulfur compounds, and is very pungent and penetrating (Hafeznia et al., 2018). Its chemical composition includes volatile essential oils (sulfur and poly sulfur) and an unstable aldehyde. It is used in traditional medicine, due to its many properties in the treatment of various diseases including bladder and kidney stones, indigestion, joint pain, flu, atherosclerosis, etc. In general, *Allium* genus is well known for its antibacterial, antioxidant, antifungal activity, and phenolic compounds (Khazaei et al., 2017).

Although studies have been conducted on the constituents of *Allium Sativum controversum* (Choobkar et al., 2021; Hafeznia et al., 2018; Kurnia et al., 2021; Sebtosheikh et al., 2020), a comprehensive study on its antioxidant properties and their use in the nonfermented sausage as a natural antioxidant has not yet been conducted. Therefore, the aim of this study was to identify phenolic and antioxidant compounds and their application in the nonfermented sausage and evaluate microbial activity in sausages.

## 2 MATERIALS AND METHODS

### 2.1 Materials

The main materials used in this study included aerial parts of *Allium sativum controversum* (Lorestan, Iran), chicken, gluten (Pishgaman shimi, Azya), sodium nitrite (BASF), potassium polyphosphate (Budenheim), ascorbic acid (Zibo), cutter (Friboi), salt and spices (Golha), sugar (Fariman), liquid oil (Oila), egg white (Telavang), chemicals (Merck), ethanol (Kiashimi), lauryl sulfate broth (LSB), yeast extract glucose chloramphenicol agar (YGC agar), plate count agar (PCA), violet red bile glucose agar (VRBG), and mannitol salt agar (MSA) media, and ringer tablet (Merck).

Different instruments used in this study included a cutter (Seydelmann), digital balance (Sartorious), oven (Tecno), incubator (P.I.T), and autoclave (Shimaz), rotary system (Heidolph), mill (Pars Khazar), digital pH meter (Metrohm), meat grinder (Panasonic Korea), furnace (Thermolab), water bath (MIC), microsampler (CAPP), and Shaker (GFL).

### 2.2 Extraction

Water and ethanol 96% are used for immersion. One hundred grams of powdered leaves of the plants was weighed by a digital balance, precisely. Then aqueous, ethanolic, and hydroalcoholic extracts were prepared individually by adding them to a conical flask containing 500 ml distilled water and ethanol 96%, sealed with parafilm, and placed on a magnetic stirrer at ambient temperature (20°C) for 72 h to obtain desirable extracts. Solvent and plant were separated by Whatman filter paper. Next, the residue was drained completely. The obtained extracts were centrifuged at 1008 *g* for 10 min. The supernatant was removed and the extracts were transferred to a rotary system where the solvent was evaporated at 80°C and the concentrated extracts were completely scraped. They were kept in a dark sterile container at 4°C (Yazdi et al., 2014).

### 2.3 Antioxidant analysis

#### 2.3.1 Determination of the total phenol content of the extracts

The total phenol content (TPC) of the extracts was measured through the Folin–Ciocalteu method. Twenty microliters of the reaction solution was mixed with 1.16 ml of distilled water and 100 μl of Folin–Ciocalteu reagent. After 1–8 min, 300 μl of Na_2_CO_3_ solution (20%) was added to the above solution. The obtained mixture was kept at 40°C for 30 min and the absorbance values of the samples were measured at 760 nm. The TPC of the extracts was expressed as gallic acid equivalent (GAE) considering the equation of the standard curve (Mazaheri et al., 2017).

#### 2.3.2 Total flavonoids measurement of the extracts

Total flavonoids measurement (TFC) was measured by the aluminum chloride colorimetric assay. As much as 0.5 ml of extract, 0.1 ml of 10% aluminum chloride, 0.1 ml potassium acetate, and 2.8 ml distilled water were mixed, kept at room temperature for 30 min and then the absorbance was read at 415 nm wavelength. TFC was reported as the milligrams per gram (mg/g) of extract. The routine was used as a standard for drawing calibration curves (Chang et al., 2002).

#### 2.3.3 DPPH radical scavenging activity

The free radical scavenging activity of herbal extracts was evaluated according to the method described by Wang et al. (2002) with some modifications (Wang et al., 2002). Briefly, equal volumes of the methanolic solution of the 1,1‐diphenyl‐2‐picrylhydrazyl (DPPH) free radical (200 μM) and of the extracts (at the various concentrations indicated) were mixed in a rapid kinetic accessory SF‐22 (Hi‐Tech Scientific). The temperature was kept at 30°C. The radical scavenging effect was continuously followed by monitoring the change of absorbance at 516 nm for 30 min, against a methanol solvent blank. The scavenging percentage was calculated according to:

$$\text{DPPH radical scavenging activity}\;\left(\%\right)=\left(A_{\text{control}}-A_{\text{sample}}\right)/A_{\text{control}}\times 100$$

where *A*_control_ is the absorbance at 516 nm of 100 μM DPPH solution without addition of the extract/fractions and *A*_sample_ is the absorbance at 516 nm of 100 μM DPPH with 5–100 μg/ml of sample. Trolox was used as a reference standard (Anggraini et al., 2019).

### 2.4 Gas chromatography mass spectrometry (GC–MS) analyses

The GC–MS analyses were carried out on a Hewlett Packard GC–MS system, model 5973, fitted with a 30 m long, cross‐linked 5% phenyl methyl siloxane (HP‐5MS 5% Phenyl Methyl Silox, Agilent 19091S‐433) (30 m × 250 μm × 0.25 μm). The velocity of the carrier gas was 1 mm/min and the temperature program of the device was set as follows. In the beginning, the temperature increased from 50 to 120°C at a rate of 4°C per minute, then to 200°C at a rate of 2°C per minute, and then to 290°C at a rate of 25°C per minute for 18 min. The injection site temperature was adjusted to 250°C. While the transmission line temperature was 250°C, the ionization voltage was 70 eV and the ionization current was set at 150 μA. Identification of each component of essential oil was done by comparing its mass spectrum with standard spectra and the Kovats Retention Index (RI) injection of normal hydrocarbons (C8–C20, C20–C40) was calculated under the same conditions as essential oil injection (Sebtosheikh et al., 2020).

### 2.5 Preparation of sausages

Sausage samples were prepared using commercial sausage formulation with 60% red meat. To do this, frozen beef was ground once by a meat grinder and again by a meat grinder with a diameter of 25 mm. The ground beef was mixed with nitrate, one‐third of the ice, spices, salt, and phosphate for 2–3 min and then oil. Half egg white, and half ice were added. After mixing for 2 min, the mentioned part of the egg white and ice were added and after several mixings, filling ingredients (flour and starch) and other additives (sugar and ascorbic acid) were added to obtain a completely emulsified paste at 4–5°C. The paste then was refrigerated for 1 h to make the stuffing process easier. A domestic meat grinder with sausage stuffing tubes was used and then the samples were cooked by steaming at 80°C for 1 h until the internal temperature reached 70–72°C. The cooked samples were cooled to 20°C (ambient temperature) under cold water and then refrigerated for 30 days. Antioxidant capacity and their microbial count were determined on the 1st, 15th, and 30th days of the storage period.

#### 2.5.1 Antimicrobial tests of sausage

##### Coliform

Coliforms test was conducted according to Iranian national standard No. 437. One milliliter of 0.1 dilutions (initial suspension) was added to the plate. Then, 15–20 ml of violet red bile broth was added. When the medium was solidified completely, the plate was incubated at 37°C for 48 h. Then, the number of grown colonies with a diameter of 0.5 mm was multiplied by the inverse of dilution factor resulting in colonies' count per gram of sample. One colony was transferred to lauryl sulfate and placed at 37°C for 24–48 h. Coliform was reported positive if gas and turbidity appeared in the Durham tube. Since the permitted coliform number in the product was 10 colonies per gram, 0.1 ml dilution was selected.

##### Mold and yeast

Mold and yeast test was conducted according to the national standard (ISIRI) No. 997. One milliliter of the initial suspension was added to the plate to which 15–20 cc of dichloran rose bengal chloramphenicol agar (DRBC agar) was added. When the medium became solid completely, the plate was incubated at 26°C for 3–5 days. After incubation, the number of grown colonies in the plate was multiplied by the inverse of dilution factor resulting in mold and yeast count per gram of sample. The allowable limit of molds and yeasts in this product is maximally 100.

##### *Staphylococcus aureus* count

*Staphylococcus aureus* count was determined according to Iranian national standard No. 1194. To investigate the contamination of samples with *S. aureus*, Baird–Parker agar was used. After dilution in peptone water medium, the sample, prepared according to the manufacturer's instruction, was plate cultured, incubated at 35–37°C for 48 h and colonies were counted.

### 2.6 Statistical analysis

All experiments were done in triplicate and the results were reported as mean ± standard error. Statistical analysis of data was performed using the SPSS, Inc., Chicago, IL software (IBM SPSS Statistics 24). Considering a normal distribution, one‐way analysis of variance (ANOVA) and Duncan's multiple range tests were initially conducted to determine any significant difference in microbiological counts at *p* < .05 (SPSS 19.0 software Package, IBM Inc.).

## 3 RESULTS AND DISCUSSION

### 3.1 Total phenolic compounds

Phenolics can be classified as simple phenols with an aromatic ring with at least one hydroxyl group and polyphenols. Polyphenolic compounds have at least two (flavonoids) and more phenolic parts (tannin) (Khalili & Ebrahimzadeh, 2015). Phenolics are secondary metabolites identified by an aromatic ring with a free hydroxyl group. They are found nearly in all parts of the plant (Ahmadi et al., 2007).

One of their most important properties is antioxidant activity making them able to lose hydrogen and free radicals (Forouzani & Askari, 2013). As shown in Table 1, the highest TPC was found for ethanolic extract of *Allium sativum controversum* showing a significant (*p* < .05) difference from other treatments. Among the different extracts, hydroalcoholic extract had the highest total phenols and the lowest total phenols were observed for ethanol extract (Table 1).

**TABLE 1:** Comparison of total phenol content (TPC) (mg gallic acid equivalent (GAE)/g) of *Allium sativum controversum* extracts

Treatment	Total phenolic content (mg GAE/g)
Aqueous extract	4.82 ± 0.20^c^
Ethanolic extract	12.24 ± 1.64^a^
Hydroalcoholic extracts	8.64 ± 0.34^b^

*Note*: Different letters indicate significant difference at the 0.05 level.

The results of this study showed that in addition to the nature of extracts, the type of solvent used for extraction and bioactive components had a significant effect on TPC. This finding is contrary to the results obtained by Nirmal et al. (2012). Their study revealed that TPC in *Solanum nigrum* L. extract obtained by ethanol was higher than in water and ether petroleum (Nirmal et al., 2012). Some studies have shown that water has a greater ability than other solvents to extract phenolic compounds (Vilkhu et al., 2008). It is worthy to note that different results of analyzed bioactive compounds of certain plants could be affected by different factors. Type and amount of essential oil or extract compounds are a function of seasonal and geographical variations, the vegetative phase, and the used part of the plant (Hashemabadi & Kaviani, 2010; Palá‐Paúl et al., 2005) as well as the type and amount of polyphenolics (Wang et al., 2002).

The concentration of phenolics in the extracts depends on factors including the solvent and method of extraction, solvent concentration, and immersion time (Kasparavičienė et al., 2013). Cheung et al. (2003) extracted phenolics of edible mushrooms by four solvents, water, methanol, ethyl acetate, and petroleum ether and reported that aqueous extract had the highest TPC (Cheung et al., 2003).

### 3.2 Total flavonoids content

Polyphenolics with two phenolic parts are known as flavonoids (Khalili & Ebrahimzadeh, 2015) having a different main structure from diphenyl propane (C_5_+ C_3_+ C_6_) with differences in the central pyran ring. They are widely spread in plant kingdoms and continue about half of 8000 identified phenols (Heim et al., 2002). They are mainly anthocyanins, anthocyanidins, flavanols, flavons, catechins, and flavanons. Flavonoids develop color in flowers and fruits and are important as secondary metabolism products in plants due to their antioxidant activity in the medicine and food industries. Mode of action of flavonoids is entrapment of free radicals and ions chelating (Khalili & Ebrahimzadeh, 2015). As flavonoids have antioxidant activity, their high content in extract suggests that the extract has great antioxidant activity in vitro (Lotito & Frei, 2004). Rutin is a glycoside flavonol as a plant metabolite with antioxidant, anti‐inflammatory, and anticancer properties. It is able to prevent vascular fragility and hypertension (Sun et al., 2011).

There were significant (*p* < .05) differences between all treatments (*p* < .05). The highest flavonoid content was observed for the aqueous extract of *Allium sativum controversum*. This finding suggests the importance of the type of extract in flavonoid content, because both extracts were extracted by water however their nature was different (Table 2). This result shows the importance of the solvent type and solvent ratios being consistent with the results obtained by (Chen et al., 2007).

**TABLE 2:** Comparison of the total flavonoids content (TFC) (milligrams of rutin equivalents (mg RE)/g) of *Allium sativum controversum* extracts

Treatment	Total flavonoid content (mg RE/g)
Aqueous extract	3.02 ± 0.02^e^
Ethanolic extract	12.04 ± 0.02^a^
Hydroalcoholic extracts	7.92 ± 0.24^c^

*Note*: Different letters indicate significant difference at the 0.05 level.

### 3.3 Antioxidant activity measurement

The ability of a compound to neutralize free radicals is expressed in IC_50_ factor representing the required sample for inhibiting 50% of free radicals. So, lower IC_50_ represents the greater antioxidant potential (a low concentration of sample can inhibit a high number of free radicals) (Vatai et al., 2009). The mode of scavenging DPPH radicals is widely used for evaluating the ability of samples to scavenge free radicals. It is a free radical with a central nitrogen atom that generates a stable molecule through reduction changing purple into yellow color. Aqueous extract of *Allium sativum controversum* showed the highest radical scavenging activity being significantly (*p* < .05) different from other treatments (Table 3).

**TABLE 3:** Comparison of radical scavenging activity (milligrams per gram [mg/g]) of *Allium sativum controversum* extracts

Treatment	Radical scavenging activity (mg/g)
Aqueous extract	11.85 ± 0.81^b^
Ethanolic extract	8.29 ± 0.10^a^
Hydroalcoholic extracts	10.09 ± 0.96^c^

*Note*: Different letters indicate significant difference at the 0.05 level.

### 3.4 GC analyses

**FIGURE 1:**
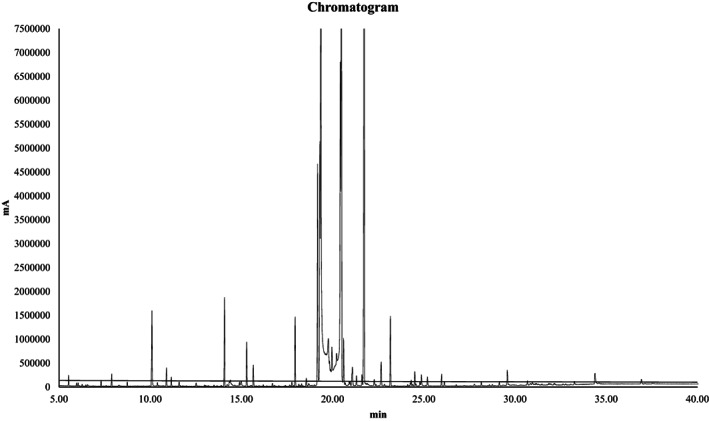
Chromatogram of the essential oil of *Allium sativum controversum*

Evaluation of the essential oil content of *Allium sativum controversum* is shown in Figure 1 and Table 4. Based on the results, 31 compounds were observed in this plant. The compounds Trisulfide, dipropyl, 1,2,4‐Trithiolane, and 3,5‐diethyl, Diallyl disulfide, in small amounts of 23%, 16%, and 11%, respectively, were the major constituents of the essential oil. Trisulfide, di‐2‐propenyl, disulfide, dipropyl, and n‐hexadecanoic acid were also present in relatively high amounts. The result is consistent with the result of Sebtosheikh et al. (2020) who evaluated essential oil from leaves and bulb of *Allium Sativum controversum* from Iran (Sebtosheikh et al., 2020). There are limited reports on the essential oil content of *Allium Sativum controversum* leaves. For example, the results of Lorigooini et al. (2014) showed that the essential oil of this species is mainly composed of sulfide compounds such as allyl methyl disulfide, methyl propyl disulfide, and dipropyl disulfide (Lorigooini et al., 2014). Yabuki et al. (2010) also identified a variety of sulfide compounds including 5 types of disulfides, 2 types of trisulfides, and 2 types of vinyldithiins (Yabuki et al., 2010).

**TABLE 4:** Chemical constituent of the essential oil of *Allium sativum controversum*

Retention time (minute)	Compound
5.5195	Tetrasiloxane, decamethy1
7.3084	AMINE, BIS[(TRIMETHYLSILYLOXY)(DIMETHYLSILYL)]‐
7.89135	Propanoic acid, 2‐methyl‐3‐[(trimethylsilyl)oxy]‐, trimethylsilyl ester $$ Isobutyric acid, 3‐trimethylsilyloxy‐trimethylsilyl ester
10.0917	Trimethylsilyl ether of glycerol
10.8862	Butanedioic acid, bis(trimethylsilyl) ester
11.1605	Propanoic acid, 2,3‐bis[(trimethylsilyl)oxy]‐, trimethylsilyl ester
14.0696	Butanedioic acid, [(trimethylsilyl) oxy]‐, bis(trimethylsilyl) ester (CAS) $$ MALIC ACID 3TMS $$ Tris(trimethylsilyl)malic acid
14.3954	2‐(2‐aminoethyl)piperidine
15.287	2,3,4‐Trihydroxybutyric acid Tetra‐TMS
15.6527	Silane, [(1‐methoxy‐1,3‐propanediyl)bis(oxy)]bis [trimethylSilane, [(1‐methoxy‐1,3‐propanediyl)bis(oxy)]bis@trimethyl]‐
17.9446	Ribitol, 1,2,3,4,5‐pentakis‐O‐(trimethylsilyl)‐
18.5561	Lyxose, tetra‐TMS‐ether
19.1734	Silane, (1,2,4,5‐cyclohexanetetrayltetraoxy)tetrakis[trimethyl]‐
19.2991	Silane, (1,2,4,5‐cyclohexanetetrayltetraoxy)tetrakis[trimethyl]‐
19.3563	.beta.‐D‐Galactopyranose, 1,2,3,4,6‐pentakis‐O‐(trimethylsilyl)‐
19.762	Silane, (1,2,4,5‐cyclohexanetetrayltetraoxy)tetrakis[trimethyl]‐
19.9564	D‐Galactose, 2,3,4,5,6‐pentakis‐O‐(trimethylsilyl)‐
20.2193	D‐Fructose, 1,3,4,5,6‐pentakis‐O‐(trimethylsilyl)‐
20.265	D‐Fructose, 1,3,4,5,6‐pentakis‐O‐(trimethylsilyl)‐
20.4308	1H‐Naphtho[2,1‐b]pyran‐6‐ol, 3‐ethenyldodecahydro‐3,4a,7,7,10a‐pentamethyl‐, [3R‐(3.alpha.,4a.beta.,6.alpha.,6a.alpha.,10a.beta.,10b.alpha.)]‐
20.4822	GlucopyranosepentaTMS $$ Glucopyranose, 1,2,3,4,6‐pentakis‐O‐(trimethylsilyl)‐, D‐
20.6022	D‐Galactose, 2,3,4,5,6‐pentakis‐O‐(trimethylsilyl)‐ (CAS) GALACTOSE‐PENTATMS
21.0823	GlucopyranosepentaTMS $$ Glucopyranose, 1,2,3,4,6‐pentakis‐O‐(trimethylsilyl)‐, D‐
21.3052	.alpha.‐D‐Galactopyranose, 1,2,3,4,6‐pentakis‐O‐(trimethylsilyl)‐ $$ Galactopyranose, 1,2,3,4,6‐pentakis‐O‐(trimethylsilyl)‐, .alpha.‐D‐
21.6081	.beta.‐D‐Glucopyranuronic acid, 1,2,3,4‐tetrakis‐O‐(trimethylsilyl)‐, trimethylsilyl ester
21.7281	Mannose, 2,3,4,5,6‐pentakis‐O‐(trimethylsilyl)‐, D‐ (CAS) $$ D‐MANNOSE 5TMS
22.2768	D‐Glucuronic acid, 2,3,4,5‐tetrakis‐O‐(trimethylsilyl)‐, trimethylsilyl ester
22.6597	Hexadecanoic acid, trimethylsilyl ester
23.1627	Myo‐Inositol, 1,2,3,4,5,6‐hexakis‐O‐(trimethylsilyl)‐ allo‐Inositol, 1,2,3,4,5,6‐hexakis‐O‐(trimethylsilyl)‐, myo‐
24.3115	Silane, [(3,7,11,15‐tetramethyl‐2‐hexadecenyl)oxy]trimethyl‐, 1‐Trimethylsiloxy‐3,7,11,15‐tetramethyl‐2‐hexadecene
24.5058	.beta.‐d‐Glucopyranoside, 2‐[[4‐(trimethylsilyl)oxy]phenyl]ethyl, tetrakis(trimethylsilyl)
24.8658	.alpha.‐Linolenic acid, trimethylsilyl ester
25.1973	Octadecanoic acid, trimethylsilyl ester
25.9746	2‐O‐Glycerol‐.alpha.‐d‐galactopyranoside, hexa‐TMS
29.5753	.alpha.‐D‐Glucopyranoside, 1,3,4,6‐tetrakis‐O‐(trimethylsilyl)‐.beta.‐D‐fructofuranosyl 2,3,4,6‐tetrakis‐O‐(trimethylsilyl)‐
34.3818	MALTOSE‐OCTA‐TMS MALTOSE 8TMS

In addition, compounds of different qualities were obtained for the first time in the leaves of *Allium sativum controversum*, which had not been reported in previous studies. For example, the compounds Naphtho [2,1‐b] pyran‐6‐ol, 3‐ethenyldodecahydro‐3,4a, 7, 7, 10 a‐pentamethyl and Glucopyranosepenta, which may indicate that different climatic conditions have different effects on the growth and the development of plants and their subsequent biochemical content. However, in general, sulfide compounds are the major compounds in this species and other dark species of *Allium*. In general, the evaluation of the essential oil profile of native species can be very important in discovering valuable medicinal and nutritional compounds, and these medicinal plants containing valuable compounds can also be used in future programs or modifications.

### 3.5 Coliforms count results

Coliforms especially *Escherichia coli* are among the most important factors in developing gastroenteritis and are indexes of water and food contamination (Ranjbar et al., 2009, 2018; Shohreh et al., 2011). Coliform contamination in meat and its products may be attributed to staff, raw materials, factory environment and equipment, during production and packaging (Sachindra et al., 2005). The highest amount of mean coliform count was found for control sample, which was significantly different from other treatments (*p* < .05).

The coliform count increased significantly from the 15th to the 30th day of the storage period. On day 1 (1 day after production), no countable colonies were observed. On the first day, the highest colony count was found for the control which was significantly (*p* < .05) higher than other treatments. On the 30th day, the highest coliform count was found for control sample due to the absence of extract and greater contamination.

**FIGURE 2:**
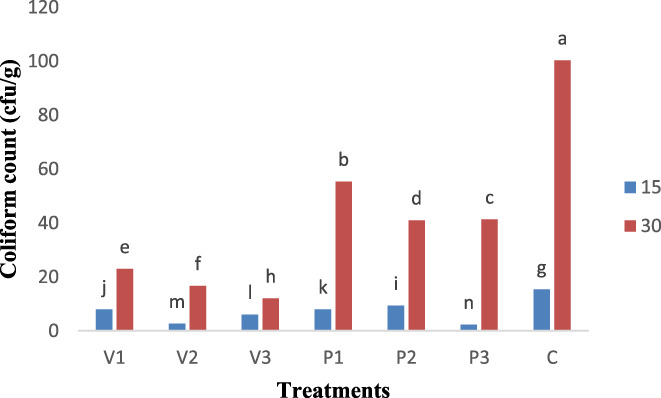
Mean comparison of coliform count (cfu/g) in sausage samples during storage period. A0.5, *Allium sativum controversum* (0.5%); A1, *Allium sativum controversum* extract (1%); A1.5, *Allium sativum controversum* extracts (1.5%); C, Control (without any extracts)

On the last day, *Allium sativum controversum* extract had a greater antimicrobial effect on coliforms and the lowest coliform count was observed for 1.5% *Allium sativum controversum* extract showing a significant (*p* < .05) difference from control and other treatments (Figure 2). Alimoradian et al. (2021) also evaluated the effect of *Carum copticum* L. and *Salvia officinalis* L. extracts on the coliform count of treated‐heat sausage during refrigerated storage and they found that the least count of coliform, mold, and yeast was found for treatment with 40 mg/kg *S. officinalis* extract (Alimoradian et al., 2021). Güngör and Gökoğlu (2010) studied the effect of plant extracts on Frankfurter and observed that microbial load in treatments containing extract decreased significantly. The main reason is the presence of antimicrobial compound (s) in extracts or essences of herbs in the formulation of meat products (Güngör & Gökoğlu, 2010).

### 3.6 Mold and yeast count

**FIGURE 3:**
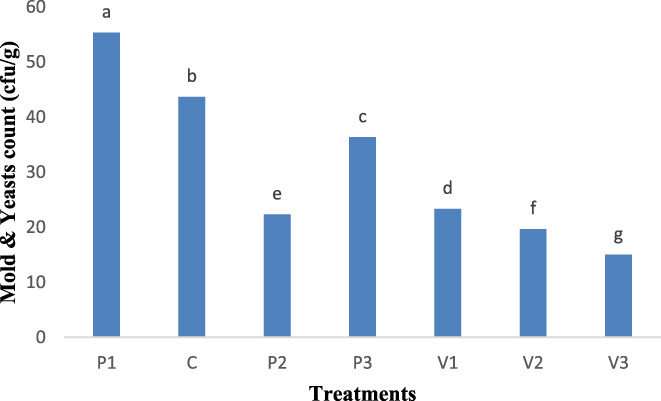
Mold and yeast content (cfu/g) in sausage samples after 30 days of storage period. A0.5, *Allium sativum controversum* (0.5%); A1, *Allium sativum controversum* extract (1%); A1.5, *Allium sativum controversum* extracts (1.5%); C, Control (Without any extracts)

Molds are aerobic microorganisms that diminish the quality of products and cause severe poisoning in some cases in a broad range of foods, especially meat products. Molds can grow and proliferate in meat products, especially dry types and even produce aflatoxin under favorable conditions including high temperatures. General judgment on moldy meat products is very difficult. Mild contaminations are mainly ignorable, however products showing severe fungal infection should be reported as unusable due to the probability of producing mycotoxins. The heating temperature when cooking has a crucial role in extending the storage life of products and controlling microbial load, mold and yeast growth (Sachindra et al., 2005). All treatments, on the last day (30) of storage period, showed significant (*p* < .05) difference. The lowest number of mold and yeast was found for 1.5% *Allium sativum controversum* extract treatment showing a significant (*p* < .05) difference from the control sample (Figure 3). Güngör and Gökoğlu (2010) compared sausage, ground meat, paste, and cooked sausage for mold and yeast and found that mold and yeast number was within the standard range because of applying a proper heat process for pasteurization (Güngör & Gökoğlu, 2010).

### 3.7 *S. aureus* count

**FIGURE 4:**
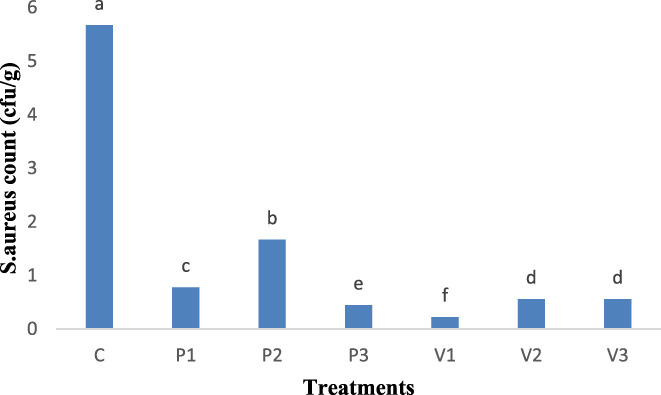
*Staphylococcus aureus* (cfu/g) in sausage samples after 30 days of storage period. A0.5, *Allium sativum controversum* (0.5%); A1, *Allium sativum controversum* extract (1%); A1.5, *Allium sativum controversum* extracts (1.5%); C, Control (without any extracts)

Antimicrobial compounds in medicinal herbs are commonly attributed to phenolic components with hydroxyl groups including thymol, carvacrol, carnosol, rutin, apigenin, terpenoid, and eugenol. Hydroxyl group attaches to the active site of enzymes preventing their metabolism. Carvacrol and its precursor, paracymene, show a synergistic relationship which is important in most medicinal herbs as initially, paracymene swells the cellular membrane facilitating more the entry of carvacrol into the cell and finally would destroy the microorganism (Crocoll et al., 2010). Figure 4 displays the comparison of mean *S. aureus* count (cfu/g) in sausage samples. The highest mean number was found for the control sample showing a significant (*p* < .05) difference. There was no significant (*p* > .05) difference between 1% and 1.5% *Allium atroviolaceum* treatments, however. The difference between other treatments and control was completely significant (*p* < .05) (Figure 4).

From the 1st to the 15th days, no countable colonies were found. On the first day, control sample had 2 cfu/g bacteria. On the 15th day, treatments containing 1% *A. atroviolaceum* had 2 cfu/g bacteria. The Control sample showed the highest *S. aureus* count. On the 30th day, all treatments had colonies of *S. aureus*, of which the highest and the lowest counts belonged to the control and *A. atroviolaceum* extract (0.5%), respectively (Figure 4). This result suggests that the extracts in the sausage formulation have antimicrobial effects.

Güngör and Gökoğlu (2010) examined the effect of herb extracts on Frankfurter and found that the treatments containing the extract had a significantly lower microbial load. The main reason was the presence of the antimicrobial compounds in extracts or essences used in meat products. Entezari et al. (2009) reported that the methanolic extract of *Echinophora platyloba* had an antibacterial effect on *S. aureus* and *Pseudomonas aeruginosa* (Entezari et al., 2009). Sharafati‐Chaleshtori et al. (2012) reported that aqueous and alcoholic extracts of *E. playloba* had an antimicrobial effect on gram‐negative and gram‐positive bacteria (Sharafati‐chaleshtori et al., 2012). Nevas et al. (2004) evaluated antibacterial properties of volatile oils of different spices on 12 bacterial species including *Escherichia coli*, *Listeria monocytogenes*, *Salmonella typhimurium*, *Clostridium botulinum*, and *Clostridium perfringens*. The results showed that the essential oil of *Mentha longifolia* and thyme had an inhibitory effect on microbial growth and a wide range of antimicrobial activity (Nevas et al., 2004).

Fernandez‐Lopez et al. (2005) studied the antibacterial activity of rosemary, orange, and lemon extracts in cooked meat and observed that rosemary extract had the greatest antioxidant activity. Crispiness and juiciness of hamburger samples were not affected by the treatments, however aroma, flavor, and total acceptance for rosemary extract were given the highest scores due to the specific aroma of rosemary and clove. According to Table 3, mold and yeast count increased over time. The greatest mold and yeast count was found for control sample. All treatments showed higher molds and yeasts on the 30th day. The additives such as sodium nitrite used in sausage inhibit the effects of extracts on mold and yeast growth. Also, there was no significant difference between the control and the two treatment combination, suggesting that the chemical additives in sausage decreased the antimicrobial effects of the extracts (Table 5).

**TABLE 5:** *Staphylococcus aureus* (cfu/g) in sausage samples after 30 days of storage

Treatments	Storage (days)		
	Day 1	Day 15	Day 30
C	3^Aa^	5.33^Ab^	8.66^Ac^
A_0.5_	0^Ba^	0^Ba^	2.66^Bb^
A_1_	0^Ba^	0^Ba^	7^Cb^
A_1.5_	0^Ba^	0^Ba^	3.66^Db^

*Note*: Capital letters indicate significant differences between storage times at the 0.05 level. Lowercase letters indicate significant differences between treatments at the 0.05 level. Control (without any extracts).

Abbreviations: A0.5, *Allium sativum controversum* (0.5%); A1, *Allium sativum controversum* extract (1%); A1.5, *Allium sativum controversum* extracts (1.5%).

## 4 CONCLUSION

The present study showed that extracts of *Allium sativum controversum*, native to Iran, could be used to improve the quality of sausage during the storage period. The presence of bioactive components such as antimicrobial and antioxidant compounds in the extracts indicates great potential in the formulation of sausage. The type of extract and solvent used both had a significant effect on total phenol, flavonoid, and radical scavenging activity. Sausage samples containing 1.0% extract showed more radical scavenging activity compared to other treatments. The results of counting *Staphylococcus aureus*, coliform, mold, and yeast in sausages after 30 days of storage also showed the antimicrobial effect of the *Allium sativum controversum* extract.

## CONFLICT OF INTEREST

There is no conflict of interest regarding the publication of this paper.
